# Cellular and clinicopathological features of the IL-33/ST2 axis in human esophageal squamous cell carcinomas

**DOI:** 10.1186/s12935-018-0700-2

**Published:** 2018-12-11

**Authors:** Guanglin Cui, Jingli Ren, Gang Xu, Zhenfeng Li, Wei Zheng, Aping Yuan

**Affiliations:** 1 Research Group of Gastrointestinal Diseases, the Second Affiliated Hospital of Zhengzhou University, Zhengzhou, Henan China; 2 Faculty of Health Science, Nord University, Campus Levanger, Levanger, Norway; 3 Department of Pathology, the Second Affiliated Hospital of Zhengzhou University, Zhengzhou, Henan China; 4 Faculty of Chemistry, Biotechnology and Food Science, Norwegian University of Life Sciences, Ås, Norway

**Keywords:** Esophagus, Carcinoma, Interleukin-33, Progression

## Abstract

**Background:**

Emerging evidence has suggested that interleukin (IL)-33 and its primary functional receptor ST2 are involved in the pathogenesis of tumorigenesis.

**Methods:**

Using immunohistochemistry (IHC) and double immunofluorescence staining, we characterized the cellular and clinicopathological features of the IL-33/ST2 axis in different compartments in human esophageal squamous cell carcinoma (ESCC) surgical specimens.

**Results:**

IHC data revealed an increased expression of IL-33-immunoreactivity (IR) and ST2-IR located in both ESCC cells and tumor stromal cells; which were associated with advanced clinicopathological features such as TNM stages and node involvement. However, the Kaplan–Meier analysis showed that densities of neither IL-33 positive nor ST2 positive cells in both the ESCC mass and stroma were associated with the overall survival rate in patients with ESCC. Double immunofluorescence staining for cellular feature analysis demonstrated that these IL-33 positive and ST2 positive cells in ESCCs were with a high proliferation rate, and IL-33-IR was frequently co-expressed with ST2-IR in both ESCC and stromal cells.

**Conclusion:**

Significant altered cellular features of the IL-33/ST2 axis in ESCCs were associated with advanced clinicopathological variables. The data suggest that the IL-33/ST2 axis might be involved in the progression of human ESCCs.

## Background

Esophageal cancer remains a highly lethal malignancy in which a frequency that varies greatly across different geographic locations [1]. Esophageal cancers are histologically subdivided into two main histologic types: adenocarcinoma and esophageal squamous cell carcinoma (ESCC). Adenocarcinoma is more common in Western countries like United States, Western and Northern Europe, whereas ESCC is more frequent seen in regions so-called “Asian esophageal cancer belt” that encompasses areas such as Iran, Kazakhstan and northern and central China [1]. Incidence of ESCC in our location (Henan province, Central China) is high and remains difficult to cure [2], primarily because of its extremely aggressive nature and frequent regional lymph node metastasis even at initial diagnosis [2, 3]. Due to this fact, much research have recently focused on mechanisms involving in ESCC tumor invasion and progression [4–7].

The precise mechanisms for ESCC progression remain unclear, though several potential mechanisms have been hypothesized and evaluated [8]. There is abundant evidence that chronic inflammation is a driving force in the majority of human malignancies including ESCC [8, 9]. Inflammatory factors released from the tumor microenvironment may lead to tumor invasion, angiogenesis and metastasis [10], which are closely associated with the prognosis in patients with ESCC [10, 11]. Thus, investigating the regulatory role of inflammatory network in the tumor microenvironment may provide not only new insights into mechanisms of ESCC progression, but also a potential therapeutic significance in the context of human cancers.

Interleukin (IL)-33 is a novel inflammatory cytokine, it plays an important role in the regulation of host immune function [12–14] and immune cell expansion [15–18], and has been associated with the development of human inflammatory diseases (IBD) [19, 20], as well as tumors [21–31]. Moreover, studies have also revealed that high expression of IL-33 is associated with disease progression and poor prognosis in diverse cancers [25, 27, 28, 32–34]. Thus, studies regarding the role of IL-33 in tumor development has attracted much attention and evidence is accumulating. Current data strongly suggest that IL-33 is involved in the pathogenesis of cancers [16, 18, 23, 31, 34–37].

Regarding mechanisms of IL-33 promoting tumorigenesis, studies have suggested that that the IL-33 may regulate the expansion and function of different T cell subsets [15, 16], ILC2s [18] and nature killer (NK) cells [17, 18], stimulate the process of angiogenesis [36, 38–40], inhibit antitumor immunity [21, 24, 41] and promote tumor cell growth [25]. For instance, recent progression has uncovered important roles of IL-33 in the stimulation of regulatory T cell (Treg) expansion and function [21, 30, 42, 43], whereas Tregs are important for the establishment and maintenance of immunosuppressive and immune tolerance in patients with cancers [44, 45]. Moreover, clinical studies have shown that increased expression of IL-33 links to the tumor invasion, metastasis [21, 25, 26, 35, 42, 46–50] and prognosis in patients with cancers [25, 34, 46]. More recently, we have demonstrated significant increased expression of IL-33 and ST2 from the colorectal precancerous (adenoma) lesion to cancerous lesion; IL-33-immunoreactivity (IR) and St2-IR are not only expressed in tumor cells, but also in surrounding stromal cells, indicating a mixt cellular source of IL-33 and ST2 in the tumor microenvironment [35]. Recently, the role of IL-33 in inducing esophageal inflammation has also been studied. Experimental evidence suggested that IL-33 contributes to the induction of chronic inflammation in esophagus and is involved in the pathogenesis of esophageal inflammatory diseases [51–53]. However, knowledge is still lacking about the role of IL-33/ST2 axis in human ESCC.

Therefore, the aim of this study was to characterize cellular features including presentation, proliferative rate, and autocrine loop of IL-33/ST2 axis presents in both ESCC and stromal cells, as well as its clinical significance.

## Methods

### Study population and tissue samples

A total of 41 patients with primary ESCC, who were diagnosed and treated in the Second Affiliated Hospital of Zhengzhou University between 2010 and 2013, were enrolled. Of these 41 patients, 26 were male and 15 were female. The age of mean at treatment for ESCCs was 56.33 years (age ranging from 32 to 76 years). Tumor location (upper/middle/lower) was 5/27/9. The pathological diagnosis and clinicopathological classification was reviewed by a senior pathologist JR from Department of Pathology according to the seventh edition of the pathologic tumor-node-metastasis (TNM) classification 2009 [54]. No patient received radiotherapy and/or chemotherapy preoperatively. No patients with Barrett’s esophagus, 65.38% (17/26) males with cigarette smoking and all females without cigarette smoking. Twenty non-tumor esophageal tissues taken from far distant locations (~ 10 cm from tumor mass) in patients with ESCC served as controls (age of mean 54.35 years, range 27–72 years; male/female: 13/7. Specimen location, upper/middle/lower: 7/3/9); six male controls with cigarette smoking and 14 without, microscopic examination showed all the controls are in normal morphology. Basic clinicopathological characteristics of patients with ESSC is summarized in Table 1. This work was approved by the local Medical Research Committee of the Second Affiliated Hospital, Zhengzhou University.

**Table 1:** Basic pathological information of ESSC patients

	N	TNM			Invasion depth		Lymph node	
		I	II	III	Muscular	All layer	+	−
ESCC	41	1	9	31	8	33	6	35

### Immunohistochemistry (IHC)

IHC for IL-33 and ST2 were performed with a Vectastain *Elite ABC* Kit (Vector Lab., Burlingame, CA, USA) according to the manufacturer’s instructions and our published methods [35, 55, 56]. The following primary antibodies were used: goat anti-IL-33 polyclonal antibody (working dilution 1:100; R&D systems, Minneapolis, MN, USA) and rabbit anti-ST2 polyclonal antibody (working dilution 1:100; Thermo Scientific., Rockford, USA). Antibodies were incubated at 4 °C overnight. 3-Amino-9-ethylcarbazole (AEC; Vector Laboratories, Burlingame, CA, USA) was used as chromogen, and slides were slightly counterstained with Mayer’s hematoxylin. Previous known colorectal adenoma/carcinoma sections shown to have IL-33 and ST2 IRs were used as positive controls to confirm the IL-33 and ST2 IRs in each series of IHCs. To exclude background staining by nonspecific antibody binding, negative controls were included using isotype-matched antibodies in each IHC test.

### Double immunofluorescence (DIF) for the examination of proliferation rate

To examine the proliferation activity of IL-33 positive and ST2 positive cells, ESCC and control sections were stained with IL-33/Ki67 (1:70; BD Pharmingen., San Jose, CA, USA) and ST2/Ki67 antibodies according to the protocol described in our previous publication [55–57]. After ESCC sections incubated with primary antibodies at 4 °C overnight, IL-33-immunoreactivity (IR) was developed with Texas red-, ST2-IR with Cy3- and Ki67-IR with FITC-conjugated secondary antibodies (all from Jackson ImmunoRearch Lab., West Grove, PA, USA). Mounted in glycerol, and viewed with confocal microscopy (LSM-700, Carl Zeiss, Jena, Germany) respectively. Colorectal adenoma/cancer sections known positive for IL-33/Ki67 and ST2/Ki67 IRs were used as positive controls. Sections with isotype-matched antibodies were used as negative controls in each DIF test and observed and photographed with a confocal microscopy (LSM-700, Carl Zeiss, Jena, Germany).

### DIF for the examination of the co-expression of IL-33 with its functional receptor ST2

To observe the co-expression of IL-33 with its functional receptor, ST2, in both ESCC and stromal cells, we therefore performed DIFs with IL-33/ST2 antibodies according to the protocol as described above; IL-33-IR was developed with Texas red- and ST2-IR with FITC-conjugated secondary antibodies. Isotype-matched negative controls were routinely performed.

### Morphometric evaluation of IHC and DIF

All the stained slides were evaluated under light microscopy and positive cells for IL-33 and ST2 in both ESCC mass and stroma were semi-quantitatively graded respectively. The numbers of cells positive for IL-33- or ST2-IRs in three well-orientated high-power fields (400×) with abundant distribution were graded as follows: (score 0), < 30% of total cell mass; (score 1), 30%**–**50% of total cell mass; (score 2), 50%**–**70% of total cell mass; (score 3), > 70% of total cell mass. The densities of Ki67/IL-33 and Ki67/ST2 double positive cells in both the epithelium and stroma in DIFs were quantified under three well-orientated middle-power fields (200×) in 10 ESCC and control sections respectively. The average values of positive cells per slide were used for statistical analysis.

### Statistical analysis

Data were present as median values plus 95% confidence interval (CI) unless otherwise stated. *P* values were evaluated by the Mann–Whitney test. The correlation between the IL-33/ST2 axis expression and clinical pathological variables was analyzed. Kaplan–Meier analysis was used to determine survival rates and differences in survival curves, the Cox proportional hazards regression model with a stepwise procedure was used to analyze the simultaneous influence of prognostic factors in available ESCC patients. *P* value < 0.05 was considered statistically significant.

## Results

### Expression of IL-33 and its functional receptor ST2 in ESCC cells and stromal cells

**Fig. 1:**
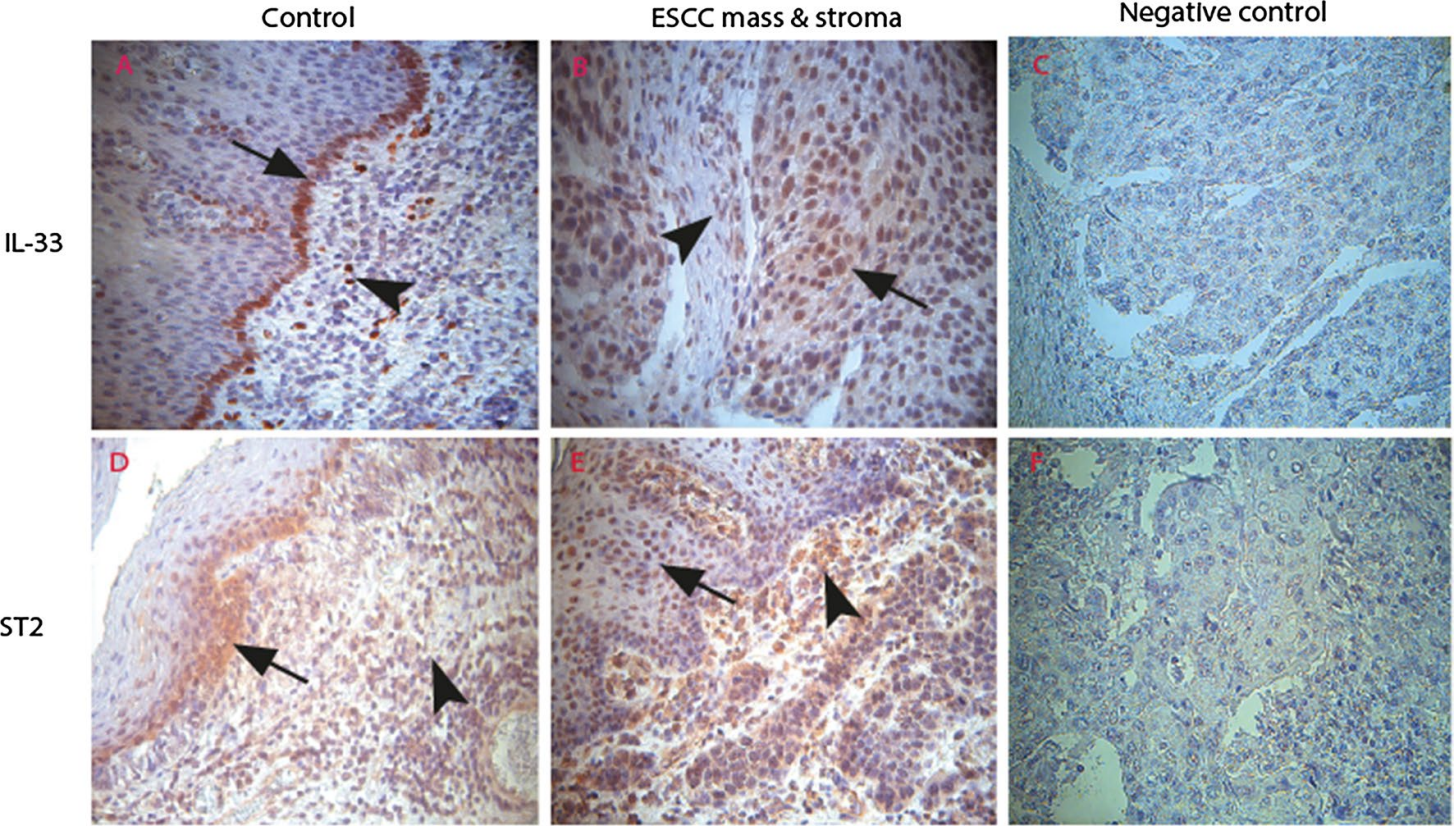
Immunohistochemical (IHC) examination of IL-33 and its functional receptor ST2 in the ESSC microenvironment. In the lamina propria of control esophageal tissues, IL-33-immunoreactivity (IR) was observed in squamous epithelium particularly in the deep layer (black arrow in **A**) and stromal cells (black arrowhead in **A**). In the stroma of ESCCs, IL-33-IR was observed in both ESCC cells (black arrow in **B**) and stromal cells (black arrowhead in **B**). Similarly, intensive ST2-IR was frequently observed in the ESCC cells (black arrow in **E**) and stroma cells (arrowhead in **E**) as compared with control sections (**D**). Both IL-33-IR and ST2-IR were not shown in isotopy-matched negative controls (**C**, **F**). (**A**–**F**: IHC, counterstained with hematoxylin, original magnification ×400)

We first examined expression of IL-33 and its functional receptor ST2 in ESCC cells and stromal cells by immunohistochemistry. As has been shown in our repost from human colorectal cancer [35], IL-33-IR was predominantly detected in nuclear of squamous epithelial cells and stromal cells in both ESCC and control tissues. In control tissues, IL-33-IR were mostly observed in the surface cells of normal epithelium (arrow pointed in Fig. 1A) and stromal cells (arrowhead pointed in Fig. 1A). The expression of IL-33-IR in ESCC cells was slightly increased (arrow pointed in Fig. 1B), but it was significantly increased in the ESCC stroma (arrowhead pointed in Fig. 1B) as compared with the controls.

ST2-IR was detected in both cytoplasm and nuclear of cells. In control tissues, it was observed in the surface cells of normal squamous epithelium (arrow pointed in Fig. 1D) and stromal cells (arrowhead pointed in Fig. 1D). Increased expression of ST2-IR in both ESCC cells (arrow pointed in Fig. 1E) and stromal cells (arrowhead pointed in Fig. 1E) was observed.

When densities of IL-33-IR and ST2-IR positive cells were semi-quantified, data confirmed above IHC observations, and showed increased density scores of IL-33-IR positive cells in the ESCC stroma and increased density scores of ST2 positive cells in both the ESCC and tumor stroma compared to the control tissue (see Table 2).

**Table 2:** Density scores of IL-33-IR and ST2-IR positive cells in the ESCC specimens

	Location	Control	ESCC	*P*
IL-33	Epithelium	2.0 (1.07–2.47)	2.0 (1.56–2.34)	> 0.05
	Stroma	1.0 (0.59–2.02)	2.0 (1.8–2.40)	< 0.05
St2	Epithelium	1.0 (0.15–2.13)	3.0 (2.00–2.60)	< 0.05
	Stroma	1.0 (0.18–2.07)	3.0 (2.27–2.68)	< 0.01

*P* values are derived from Mann-Whitney tests

### The correlation between IL-33/ST2 expression and various clinicopathological parameters and prognosis in patients with ESCC

Subsequently, the correlation between density scores of IL-33/ST2 positive cells and clinicopathological variables such as TNM pathological classification, invasion depth and node involvement were investigated. Results showed that density scores of IL-33-IR positive and ST2-IR positive cells in both the ESCC mass and stroma correlated with TNM stages (Table 3). Patients with earlier stages (stage I or II) had lower density scores of either IL-33-IR positive or ST2-IR positive cells than those with advanced stages (see Table 3, all P values were from Mann–Whitney tests). Density scores of IL-33-IR positive cells in the ESCC stroma and ST2-IR positive cells in both the ESCC mass and stroma correlated with positive node involvement (see Table 3). In addition, density scores of ST2-IR positive stromal cells in the ESCC showed a positive correlation with tumor invasion depth, ESCC patients with all layer invasion had a higher density score of ST2-IR positive stromal cells than those with only muscular invasion (see Table 3).

**Table 3:** Correlation between density scores of IL-33-IR/ST2-IR positive cells and TNM stage, node involvement and invasion depth in patients with ESCC

Parameters	IL-33-IR positive cell density scores		ST2-IR positive cell density scores	
	ESCC	Stroma	ESCC	Stroma
TNM stage				
I + II	2.0 (0.87–2.53)	1.5 (0.90–1.90)	2.0 (1.27–2.53)	2.0 (1.52–2.48)
III	3.0 (2.30–2.77)	3.0 (2.14–2.72)	3.0 (2.08–2.78)	3.0 (2.41–2.82)
P value	< 0.05	< 0.01	< 0.05	< 0.05
Node involvement				
Positive	2.5 (1.93–3.08)	3.0 (2.41–3.26)	3.0 (2.13–3.21)	2.0 (1.93–3.08)
Negative	3.0 (2.02–2.61)	2.0 (1.73–2.32)	3.0 (1.89–2.58)	2.0 (2.24–2.70)
P value	> 0.05	< 0.05	< 0.05	< 0.05
Invasion depth				
Muscular	3.0 (1.49–3.26)	1.5 (1.00–2.25)	2.5 (1.51–2.99)	2.0 (1.55–2.45)
All layer	2.0 (1.39–2.29)	2.5 (1.88–2.56)	3.0 (1.99–2.69)	3.0 (2.37–2.82)
P value	> 0.05	> 0.05	> 0.05	< 0.05

*P* values were derived from Mann–Whitney tests

**Fig. 2:**
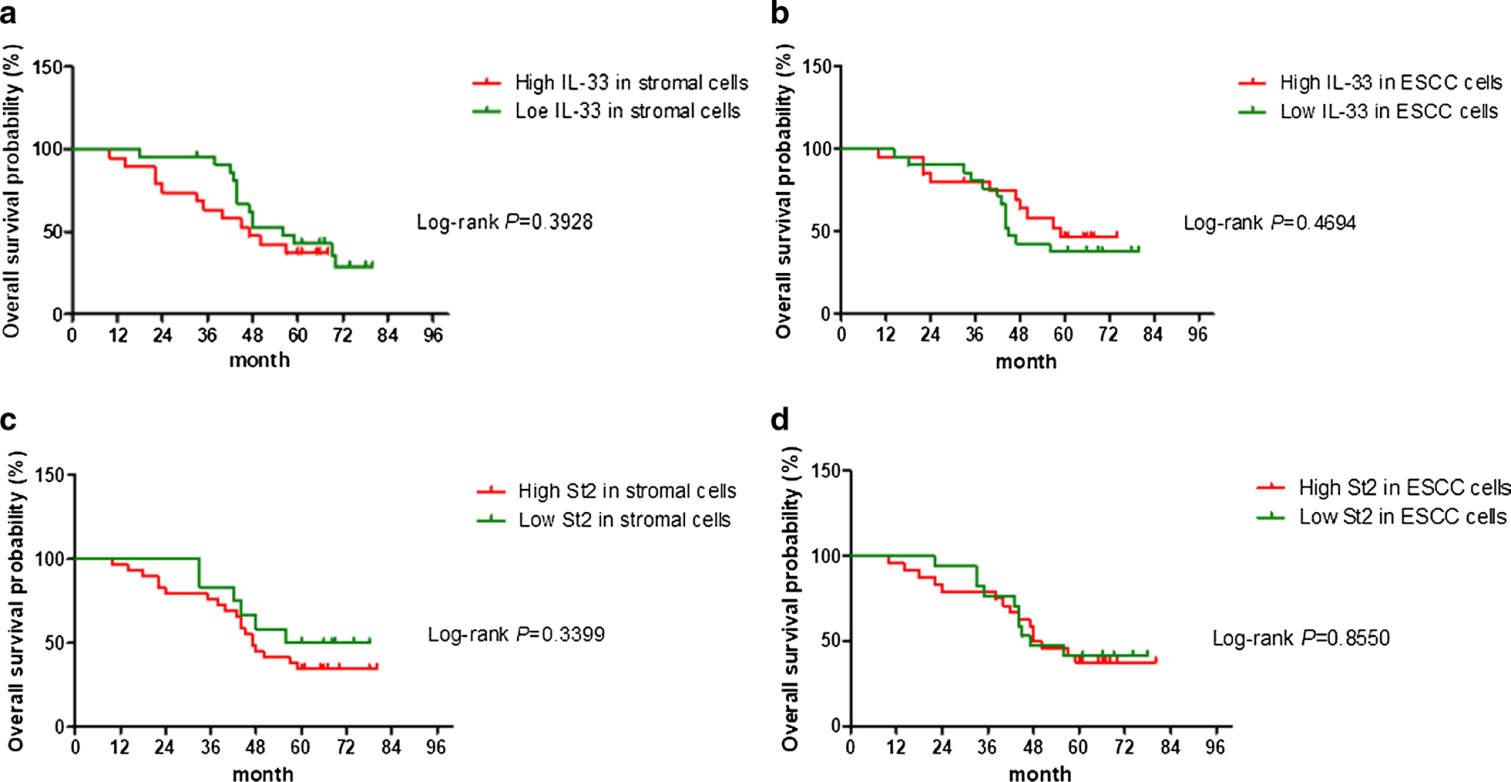
The Kaplan–Meier analysis of overall survival differences among ESCC patients with different densities of IL-33-IR and ST2-IR positive cells in the tumor stroma. Kaplan–Meier analysis revealed that densities of IL-33-IR positive (**a**, **b**) and ST2-IR positive cells (**c**, **d**) in both ESCC mass and stroma do not predicate the overall survival rate in patients with ESCC (all *P* values determined by log-rank tests)

Finally, Kaplan–Meier survival curves revealed that density scores of neither IL-33-IR positive (Fig. 2a, b) nor ST2-IR positive cells (Fig. 2c, d) in both the ESCC mass and stroma correlated with the overall survival in patients with ESCC.

### Proliferation activity of IL-33 positive and ST2 positive cells in the ESCC

**Fig. 3:**
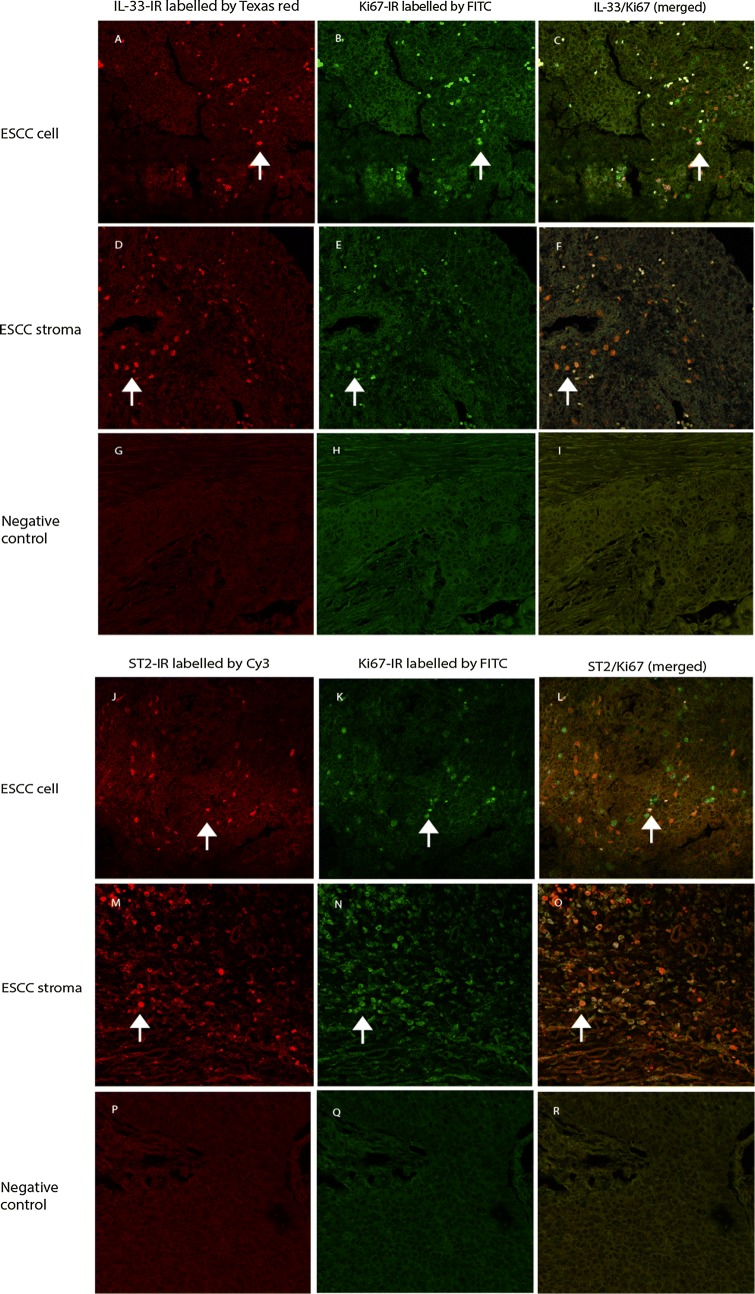
Double immunofluorescence (DIF) staining with confocal microscopy to evaluate proliferative activity of IL-33-IR and ST2-IR positive cells in the ESCC mass and stroma. DIFs images revealed that IL-33-IR (labelled by Texas red, red cells) in the ESCC mass (**A**) and stroma (**D**) was frequently co-localized (merged images in **C**, **F**) with a high rate of Ki67-IR (labelled by FITC, green cells in **B**, **E**). ST2-IR (labelled by Cy3, red cells) in the ESCC mass (**J**) and stroma (**M**) was co-localized (merged images in **L**, **O**) with a high rate of Ki67-IR (labelled by FITC, green cells in **K**, **N**). IRs for targeted proteins were not shown in both isotopy-matched negative controls for each DIF (see **G**–**I**, and **P**–**R** respectively). (**A**–**R**: DIFs, original magnification ×200; counterstaining was not applied)

The analysis demonstrated that both IL-33-IR positive (Fig. 3A–F) or ST2-IR positive (Fig. 3G–L) ESCC tumor cells and stromal cells had a high proliferative rate. Quantitative data showed that densities of Ki67/IL-33 and Ki67/ST2 positive cells were increased in both ESCC and stromal cells as compared with controls (Ki67/IL-33 in ESCC vs. control: 17.0 (13.98–20.22) vs. 11.50 (10.28–14.92), P < 0.05; Ki67/IL-33 in stroma vs. control: 23.00 (18.09–26.51) vs. 17.50 (13.42–20.38), P < 0.05. Ki67/ST2 in ESCC vs. control: 14.50 (10.48–17.72) vs. 9.02 (6.06–12.14), P < 0.05; Ki67/ST2 in stroma vs. control: 18.0 (15.31–21.49) vs. 10.0 (7.64–13.56), P < 0.01. P values were obtained from the Mann–Whitney test).

### IL-33 and its functional receptor ST2 are expressed in same cells in the ESCC

**Fig. 4:**
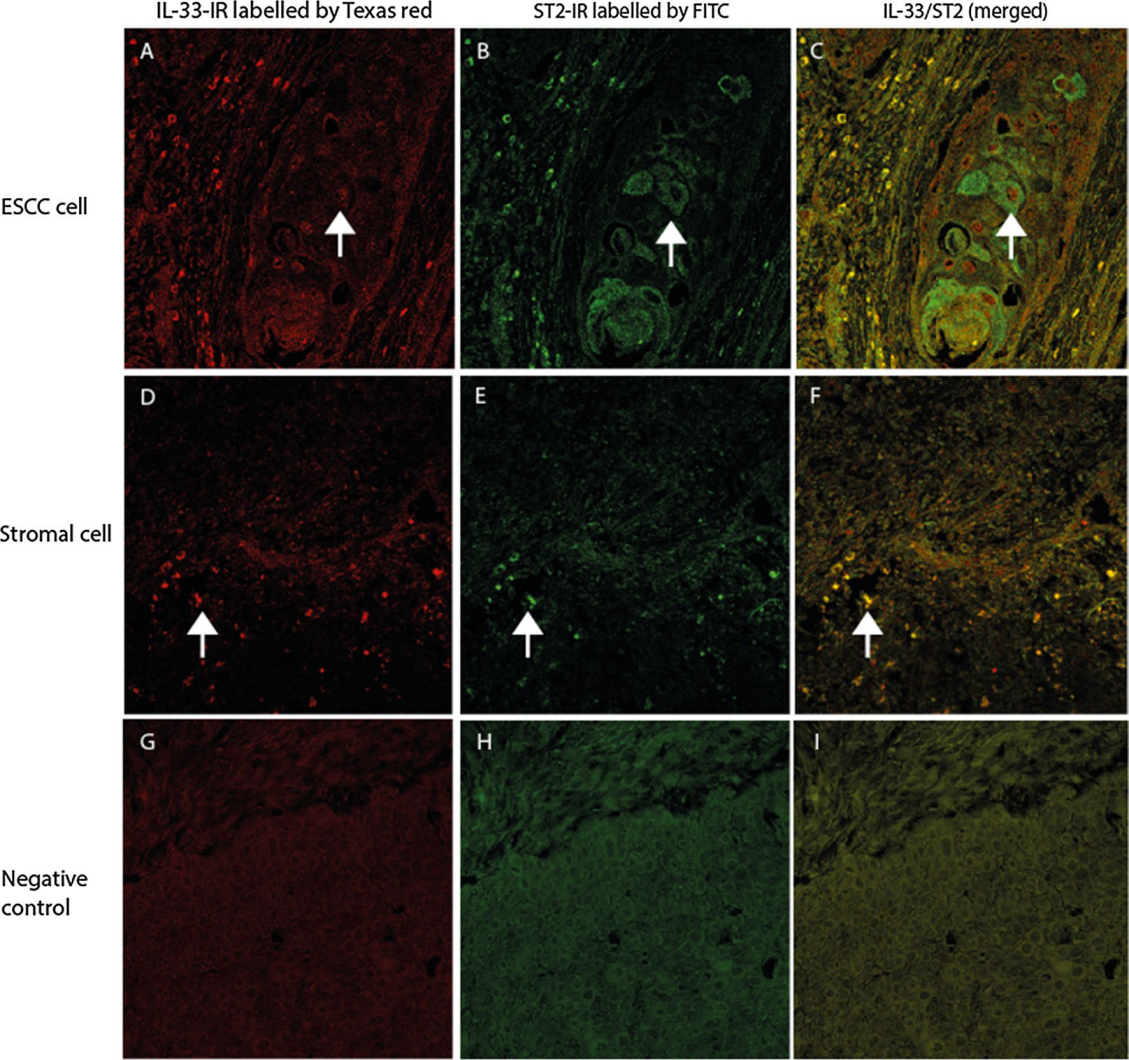
Double immunofluorescence (DIF) staining with confocal microscopy to evaluate the co-expression of IL-33 with its receptor, ST2, in the ESCC section. DIF images showed that IL-33-IR (labelled by Texas red, red cells in **A**, **D**) was frequently co-localized (merged images in **C**, **F**) with ST2-IR (labelled by FITC, green cells in **B**, **E**) in ESCC cells (merged image in **C**) and stromal cells (merged image in **F**). IRs for targeted proteins were not shown in isotopy-matched negative controls (**G**–**I**). (**A**–**I**: DIFs, original magnification ×200; counterstaining was not applied)

DIF results indicated that some of ESCC cells (Fig. 4A–C) and stromal cells (Fig. 4D–F) were positive for both IL-33-IR and ST2-IR, implying that these cells could either be the cellular sources or target for IL-33 and ST2 in the ESCC.

## Discussion

In this study, we investigated cellular features of IL-33 and its functional receptor, ST2, expressions and evaluated its clinicopathological significance in patients with ESCC. We found that expressions of IL-33 and ST2 were significantly increased in both ESCC cells and stromal cells with a high proliferation rate. Density scores of either IL-33-IR or ST2-IR positive cells in the ESCC stroma correlated with advanced clinicopathological variables i.e. TNM stage, node involvement and invasion depth, which may suggest that the IL-33/ST2 axis is involved in the progression of human ESCC. To best of our literature knowledge, the current study is the first study to characterize the cellular and clinicopathological features of the IL-33/ST2 axis in patients with ESCCs.

Although there has been considerable interest in the essential role of IL-33/ST2 axis in tumorigenesis [18, 30, 31], studies have provided contradictory results with both pro-tumor and anti-tumor effect reported [29–31, 58]. It appears likely that such different roles might be related to the types of tumor studied and the models used [29–31, 58]. Thus, the role of IL-33/ST2 axis in human tumors has been the subject of controversy, because of its complexity and context dependence. In this study, we were able to demonstrate that both IL-33-IR and its functional receptor ST2-IR were highly expressed in ESCC cells and stromal cells, where most of the cells were actively proliferating. Taken together with previous finding that IL-33 is an early alarm signal rapidly released from producing cells upon cellular damage or cellular stress [59], our findings may suggest that such increased expression of the IL-33/ST2 axis in the ESCC might be reflect the an active IL-33/ST2 immune reaction during esophageal tumorigenesis.

It has been recently reported that the expression of IL-33 is associated with clinicopathological variables in certain types of cancers [25, 34, 46]. We therefore analyzed the clinicopathological significance of IL-33 and ST2 expressions in different compartment elements in the ESCC. Results did show that density scores of IL-33-IR positive or ST2-IR positive cells in the ESCC stroma were closely associated advanced clinicopathological variables i.e. TNM stages and invasion depth, these data suggest that the IL-33/ST2 axis is involved in the progression of human ESCC. However, Kaplan–Meier survival curve analysis showed that density scores of neither IL-33-IR positive nor ST2-IR positive cells in different compartments were associated with overall survival in patients with ESCC. This may reflect the fact that a complex network with multiple elements such as growth factors, immune function, angiogenesis and stroma response, rather than a sole cytokine determines the prognosis of human tumors [9].

Regarding the mechanisms of IL-33/ST2 axis promoting tumorigenesis, increasing evidence suggests that one of the possibilities is through the activation of tumor stroma [30, 60, 61]. Our double immunofluorescence images showed that both IL-33-IR and ST2-IR positive ESCC cells and stromal cells showed a high proliferative rate, which indicates an active IL-33/ST2 response occurred in the ESCC microenvironment. Furthermore, we found that IL-33- and its receptor ST2 IRs were observed in both ESCC cells and stromal cells, which confirmed that these cells could be either the cellular sources or target for IL-33. Whether there is an autocrine or paracrine action way need to be explore in vitro, which might help to design novel translational-targeted agents in the future.

## Conclusion

Data present in this study add an advance in our understanding of the role of IL-33/ST2 axis in ESCC progression, by demonstrating high expression levels of IL-33 and its primary functional receptor ST2 in ESCC and stromal cells, which are associated with advanced clinicopathological variables. This new work also supports exploration of designing novel translational-targeted agents. Together with other recent researches of the IL-33/ST2 axis in human cancers [35], our work supports the hypothesis that the IL-33/ST2 axis may play an important role in ESCC progression. Although more studies are required involving additional work to explore the exact mechanisms of the IL-33/ST2 axis in ESCC progression, the weight of evidence supports a contributing role for IL-33 and its receptor ST2 to the stromal activation of ESCC, which may result in the development of new therapeutic targets for ESCC treatment.

## Abbreviations

ESCC: esophageal squamous cell carcinoma
IL: interleukin
IHC: immunohistochemistry
DIF: double immunofluorescence
FITC: fluorescein
CI: confidence interval
TNM: tumor/node/metastasis
